# Alteration of plasma metabolites associated with chemoradiosensitivity in esophageal squamous cell carcinoma via untargeted metabolomics approach

**DOI:** 10.1186/s12885-020-07336-9

**Published:** 2020-09-02

**Authors:** Yaowen Zhang, Jianpo Wang, Ningtao Dai, Peng Han, Jian Li, Jiangman Zhao, Weilan Yuan, Jiahuan Zhou, Fuyou Zhou

**Affiliations:** 1 Anyang Cancer Hospital, The 4th Affiliated Hospital of Henan University of Science and Technology, No.1 Huanbin North Road, Anyang, 455000 Henan Province China; 2 Shanghai Zhangjiang Institue of Medical Innovation, Shanghai Biotecan Pharmaceuticals Co., Ltd., 180 Zhangheng Road, Shanghai, 201204 China

**Keywords:** Chemoradiosensitivity, Esophageal squamous cell carcinoma, Metabolomics, Neoadjuvant therapy, Untargeted metabolomics analysis

## Abstract

**Background:**

To investigate the differences in plasma metabolomic characteristics between pathological complete response (pCR) and non-pCR patients and identify biomarker candidates for predicting the response to neoadjuvant chemoradiotherapy (nCRT) in esophageal squamous cell carcinoma (ESCC).

**Methods:**

A total of 46 ESCC patients were included in this study. Gas chromatography time-of- flight mass spectrometry (GC-TOF/MS) technology was applied to detect the plasma samples collected before nCRT via untargeted metabolomics analysis.

**Results:**

Five differentially expressed metabolites (out of 109) was found in plasma between pCR and non-pCR groups. Compared with non-pCR group, isocitric acid (*p* = 0.0129), linoleic acid (*p* = 0.0137), citric acid (*p* = 0.0473) were upregulated, while L-histidine (*p* = 0.0155), 3′4 dihydroxyhydrocinnamic acid (*p* = 0.0339) were downregulated in the pCR plasma samples. Pathway analyses unveiled that citrate cycle (TCA cycle), glyoxylate and dicarboxylate metabolic pathway were associated with ESCC chemoradiosensitivity.

**Conclusion:**

The present study provided supporting evidence that GC-TOF/MS based metabolomics approach allowed identification of metabolite differences between pCR and non-pCR patients in plasma levels, and the systemic metabolic status of patients may reflect the response of ESCC patient to neoadjuvant chemoradiotherapy.

## Background

Esophageal cancer (EC) as an aggressive malignant tumor, is the sixth leading cause of cancer death globally [1]. Over 50% of all EC-related deaths occur in China where esophageal squamous cell carcinoma (ESCC) is the predominant histologic subtype [2]. Surgery is the primary treatment of esophageal cancer, especially for patients with early stage [3, 4]. But most esophageal cancer cases has progressed to advanced stage before they are finally diagnosed [5]. Neoadjuvant chemoradiotherapy (nCRT) has been considered as a promising therapy strategy for patients with stage II or III esophageal cancer. Several studies have shown that neoadjuvant chemoradiotherapy plus surgery contributes to improved local control, progression free survival, and overall survival compared with surgery alone [6–8]. However, not all the EC patients could benefit from nCRT and poor responders have to experience severe toxicity and impaired quality of life [9, 10]. Moreover, the outcomes of non-responders were found to be worse than those underwent primary resection [11]. Hence, it is essential to find a reliable marker of chemoradiosensitivity in esophageal cancer to avoid wasting valuable time and obtain a more favorable prognosis for patients.

Metabolomics have been widely applied in diagnosis and biomarker screening study on various disease, including cancer [12, 13]. Since metabolites represent the end products of biochemical processes, it is closely linked to the overall physiopathological status of an individual [14]. It has been discovered that alterations of metabolites in the biofluids (serum, plasma, and urine) are related to prognosis [15], recurrence [16], treatment response [17] in cancer patients. So far, metabolomic studies on EC have been performed to identify differential metabolite markers between patients and controls [18]. In addition, multiple metabolites have been found to be strongly associated with the degree of tumor progression through metabolomics-based methods [19, 20]. As for the biomarker screening for chemoradiosensitivity in ESCC, one research with small sample size revealed that serum levels of several metabolites differed significantly between the pathological complete response (pCR) group and non-pCR group [21]. However, no other external validation was provided.

In the present study, we aim to investigate the differences in plasma metabolomic characteristics between pCR and non-pCR patients and identify biomarker candidates for predicting the response to neoadjuvant chemoradiotherapy (nCRT) in esophageal squamous cell carcinoma (ESCC). We used gas chromatography time-of-flight mass spectrometry (GC-TOF/MS) technology which is more conducive to the rapid detection of complex samples analysis for untargeted metabolic profiling. The results showed that five metabolites demonstrated differences between pCR and non-pCR patients in the plasma collected before the onset of neoadjuvant therapy. And pathway analyses unveiled that citrate cycle, glyoxylate and dicarboxylate metabolic pathway were associated between pCR and non-pCR groups. This study provided supporting evidence that GC-TOF/MS based metabolomics approach allowed identification of metabolite differences between pCR and non-pCR patients in plasma levels, and the systemic metabolic status of patients may reflect the response of ESCC to neoadjuvant chemoradiotherapy.

## Methods

### Sample collection

The study included plasma samples from 46 stage II–III esophageal cancer patients who were prospectively selected at the Anyang Cancer Hospital (Henan, China) between June 2017 and April 2019. All patients had been pathologically diagnosed esophageal squamous cell carcinoma. The neoadjuvant chemoradiotherapy (nCRT) consisted of radiotherapy (total radiation dose: 45Gy, 1.8Gy/day, 25 fractions) and concurrent chemotherapy with paclitaxel (135–150 mg/m^2^) plus cisplatin (50–75 mg/m^2^) every 21 days for two cycles. 4–6 weeks after completion of nCRT, patients underwent surgery. Clinical stages and pathological stages were determined according to the eighth edition of the American Joint Committee on Cancer tumor-node-metastasis (TNM) staging criteria [22]. All pathology slides were reviewed by a pathologist to determine the pathologic response. pCR was defined as no evidence of viable tumor cells in all specimens, including the primary site and lymph nodes [23]. Samples were collected just before the onset of neoadjuvant therapy and kept frozen and stored at − 80 °C for further analysis. This study was approved by the Ethics Committee of Anyang Cancer Hospital.

### Sample preparation for metabolomic analysis

The untargeted metabolomics profiling was implemented on XploreMET platform (Metabo-Profile, Shanghai, China). The sample preparation was conducted as their published methods with minor modifications [24, 25]. In brief, the plasma samples were centrifuged at 3000×*g* and 4 °C for 5 min (Microfuge 20R, Beckman Coulter, Inc., Indianapolis, IN, USA) after thawing to separate debris or a lipid layer. Metabolites were extracted from plasma samples (50 μL) with 10 μL of internal standard (0.5 mM 4-Chlorophenylalanine) and 175 μL of pre-chilled methanol: chloroform (3:1) followed by centrifugation at 14, 000×g for 20 min at 4 °C. Then each 200 μL of the supernatant was transferred into an autosampler vial (Agilent Technologies, Foster City, CA, USA). The resting supernatant from each sample was pooled to prepare quality control samples. Following solvent evaporation and lyophilization, the dried samples were derivatized with 50 μL of methoxyamine (20 mg/ml in pyridine) for 2 h, followed by silylanization with 50 μL of MSTFA (1% TMCS) for 1 h prior to injection. Above two steps were performed by a robotic multipurpose sample MPS2 with dual heads (Gerstel, Muehlheim, Germany).

### Metabolomic analysis

The GC-TOF/MS analysis was performed using a time-of-flight mass spectrometry (GC-TOF/MS) system (Pegasus HT, Leco Corp., St. Joseph, MO,USA),which consists of an Agilent 7890B gas chromatography and a Gerstel multipurpose sample MPS2 with dual heads (Gerstel, Muehlheim, Germany). As described previously [26], DB-5MS GC column (30 m × 250 μm i.d., 0.25-μm film thickness; Restek corporation, Bellefonte, PA, USA) was chosen for separation. Helium was used as the carrier gas at a steady flow rate of 1.0 mL/min. The temperature of transfer interface and injection were both 270 °C. The source temperature was set as 220 °C. The measurements were taken using electron impact ionization (70 eV) in the full scan mode (m/z 50–500).

### Data processing

XploreMET (v3.0, Metabo-Profile, Shanghai, China) was used to process the raw data generated by GC-TOF/MS. The data processing includes baseline denosing and smoothing, peak picking and deconvultion, creating reference database from the pooled QC samples, metabolite signal alignment, missing value correction and imputation, and QC correction as previously reported [27]. Metabolites were identified by comparing both retention index and mass spectral data with JiaLibTM metabolite database. Each data set was converted into comparable data vectors for statistical analysis. The metabolites with t test or U test P <0.05 by unidimensional analysis were considered as differentially expressed between pCR and non-pCR groups.

### Pathway analyses

To further investigate the metabolic pathways involved in the chemoradiosensitivity in ESCC, the differential metabolites were annotated with Kyoto Encyclopedia of Genes and Genomes (KEGG, http://www.genome.jp/kegg/) and Human Metabolome Database (HMDB, http://www.hmdb.ca/). MetaboAnalyst 4.0 (http://www. metaboanalyst.ca/MetaboAnalyst/) were then applied to analyze the data by R software (v3.4.3, GitHub). The iPath 3.0 (http://pathways.embl.de/) was used to show the metabolic network of the differential metabolites and altered metabolic pathways in KEGG general metabolic pathway.

### Statistical analysis

All measurements were mean-centered and scaled by the standard deviation of the observed measurements. Fisher’s exact probability test, the Student’s t-test, or the Mann–Whitney U-test was used to evaluate the statistical significance of differences. The Mann–Whitney U test was utilized for non-normal distribution data and the Student’s t-test was applied for normal distribution data. Fisher’s exact tests for categorical variables. The area under the receiver operating characteristic (ROC) curve (AUC) was performed to assess the feasibility of using the plasma levels of particular metabolites as predictive biomarkers. The data was randomly split into a training set (data from 36 patients) and a test set (data from 10 patients). Predictions were made on the test set based on the Gaussian Naive Bayes model trained in the training set. *P* < 0.05 was considered statistically significant.

## Results

### Patients & treatment outcomes

A total of 46 patients were included in this study. The age of all subjects ranged from 50 to 84 years, including 30 men and 16 women. According to the American Joint Committee on Cancer tumor-node-metastasis (TNM) staging criteria (the eighth edition), the pre-treatment clinical stages of these 46 subjects were II (*n* = 4) and III (*n* = 42), respectively. After nCRT, 23 patients achieved pCR through the examination of surgical pathology specimen, while the remaining 23 patients were identified into non-pCR group. The baseline characteristics of the ESCC patients and treatment outcomes are shown in Table 1. According to Fisher’s exact probability test, tumor location and clinical stage are significantly different between the pCR and non-pCR groups. There were no significant differences in gender, age, smoking and alcohol consumption between the two groups.

**Table 1:** Clinical characteristics of the patients

Clinical characteristics	Case	Histological response		*P* values
	*n* = 46	non-pCR (*n* = 23)	pCR (*n* = 23)	
Gender				0.758
Male	30	16	14	
Female	16	7	9	
Age				1.000
<65	19	9	10	
≥ 65	27	14	13	
Tumor location				0.041*
Cervical	2	2	0	
Lt	7	5	2	
Mt	23	13	10	
Ut	14	3	11	
TNM stage				0.028*
II	4	0	4	
III	42	23	19	
Smoking				0.136
Yes	20	13	7	
No	26	10	16	
Drinking				1.000
Yes	16	8	8	
No	30	15	15	

*p< 0.05

### Altered metabolites in plasma among pCR and non- pCR patients

**Fig. 1:**
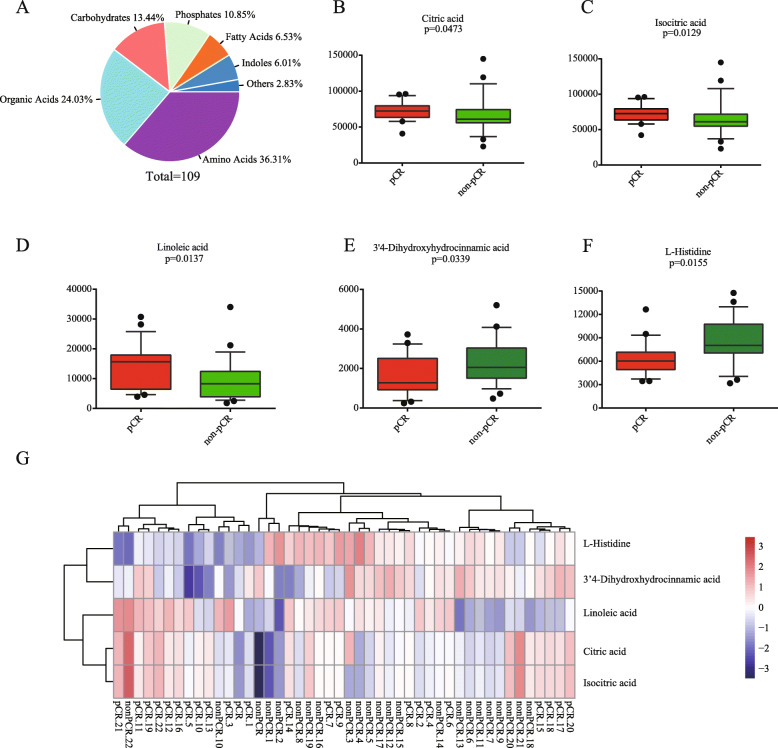
Altered metabolites in plasma among pCR and non-pCR patients. **a** Pie chart illustrating the abundance ratio of different classes of metabolites detected by untargeted metabolic profiling in plasma samples from ESCC patients. **b**-**f** Five identified metabolites that differed significantly between pCR and non-pCR groups using unidimensional analysis. **g** Hierarchical clustering of significantly different metabolites. The tree structure on the left side represents the clustering relationships of each metabolite, and the tree structure at the top represents the clustering relationships of each sample

A total of 109 metabolites of different classes (Amino Acids, Organic Acids, Carbohydrates, Phosphates, Fatty Acids, Indoles) were detected in the plasma samples from 46 subjects using untargeted metabolomics analysis (Fig. 1a and Supplementary List 1). To further determine whether the metabolite footprints in plasma differed between pCR and non-pCR subjects, unidimensional analysis was used (Student’s t-test or Mann–Whitney U test was selected according to the data) to obtain the differential metabolites between the two groups. We found five differentially expressed metabolites (out of 109) in plasma levels between pCR and non-pCR groups. Compared with non-pCR group, isocitric acid, linoleic acid, citric acid were upregulated (*p* = 0.0129, 0.0137 and 0.0473, respectively), and L-histidine, 3′4-Dihydroxyhydrocinnamic acid were downregulated (*p* = 0. 0.0155 and 0.0339, respectively) in the pCR plasma samples (Fig. 1b-f). In order to display the relationships among samples and differences in expression patterns more intuitively, we also conducted the bidirectional clustering analysis of each samples and the significantly different metabolites. As the results showed that the samples in two groups can be distinguished, and metabolites between two groups existed difference (Fig. 1g).

**Fig. 2:**
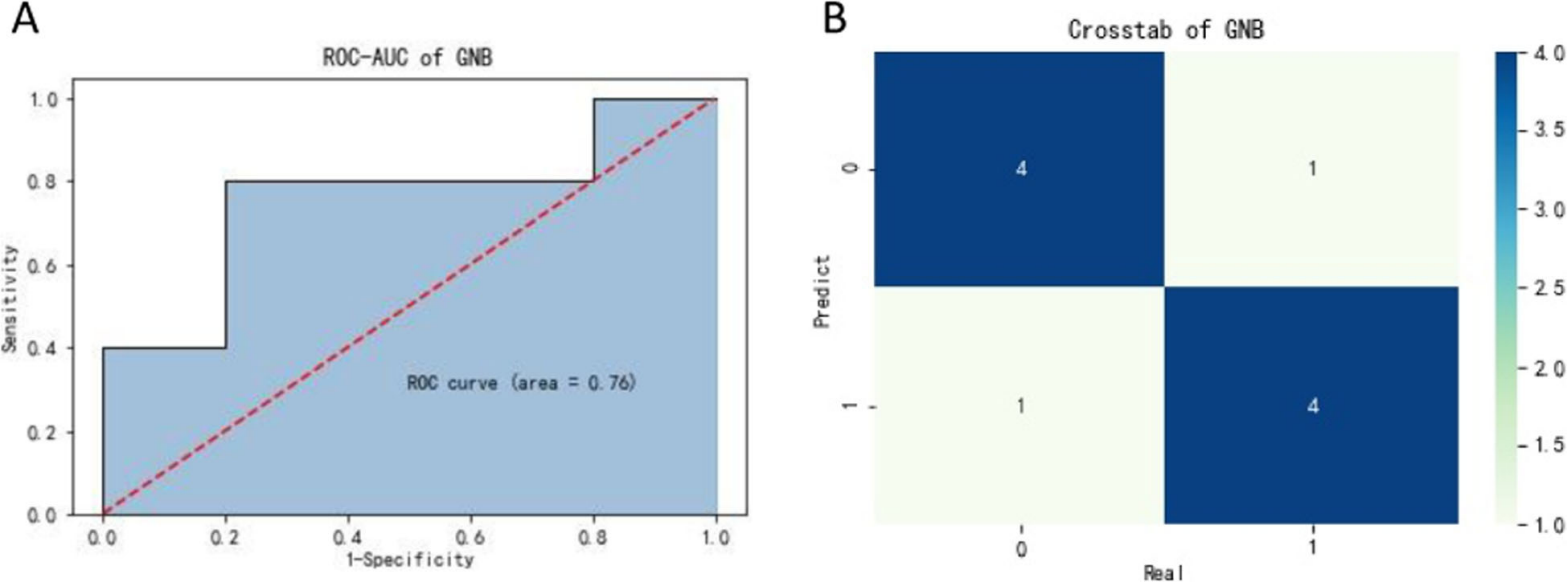
Evaluation of the five differential metabolites in predictive precision. **a** ROC curve based on the five differentially expressed plasma metabolites. **b** Confusion matrix showing classification results (80% overall correct assignment)

Furthermore, ROC analysis was applied to explore whether the five differential metabolites could be converted into an approach for predicting nCRT response. As the ROC curve performed by Gaussian Naive Bayes model showed that the area under the curve (AUC) value was 0.76 (Fig. 2), which implied a good ability in predicting the response of pCR and non-pCR of esophageal cancer patients by these five metabolites.

### Metabolic pathway analysis

**Fig. 3:**
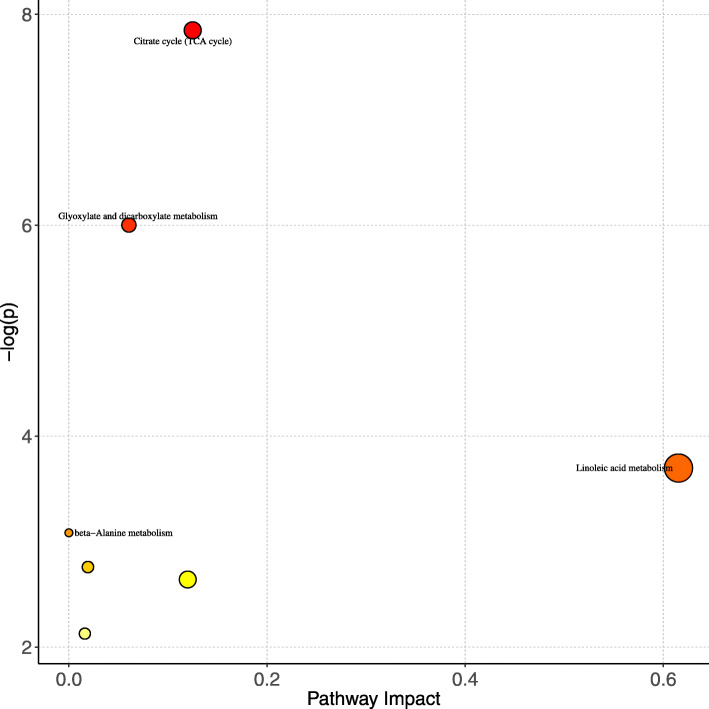
Pathway analysis of the differential metabolites between the pCR and non-pCR groups. The altered pathway of ESCC chemoradiosensitivity-associated metabolites. The color and size of each circle is based on P value and pathway impact value respectively

To further explore the metabolic pathways related to above ESCC chemoradiosensitivity-associated metabolites, MetaboAnalyst was used to indicate the metabolic pathways connected with these five metabolites. Through pathway analysis based on KEGG database, two pathways namely citrate cycle (TCA cycle), glyoxylate and dicarboxylate metabolism were altered in pCR patients (Fig. 3 and Table 2). The overall metabolic network of the altered metabolites and metabolic pathways is shown in Supplementary Figure 1.

**Table 2:** Pathway analysis of metabolite changes

	Total In Pathway	Hits	***p*** Value	Impact	Enriched Compounds
Citrate cycle (TCA cycle)	20	2	0.00038978	0.125	Citric acid Isocitric acid
Glyoxylate and dicarboxylate metabolism	50	2	0.0024713	0.0606	Citric acid Isocitric acid
Linoleic acid metabolism	15	1	0.024711	0.61538	Linoleic acid
beta-Alanine metabolism	28	1	0.045753	0	L-Histidine
Nitrogen metabolism	39	1	0.063291	0.01923	L-Histidine
Histidine metabolism	44	1	0.071183	0.12	L-Histidine
Aminoacyl-tRNA biosynthesis	75	1	0.119	0.01613	L-Histidine

## Discussion

Although the benefit of nCRT has been demonstrated in multiple trials, only approximately 20–25% of EC patients showed complete response, and some even developed resistance to currently used therapy [28]. Chemoradiotherapy resistance is a considerable obstacle to the effective treatment of esophageal cancer. Unfortunately, we have not yet identified specific biomarkers in blood which would reliably and accurately predict response to nCRT.

Metabolism alterations in cancer are well-documented. Metabolomics as a new high throughput technology provides a broad field for finding metabolites as potential biomarkers in body fluids for diagnosis, treatment and prognosis of cancer. Gas chromatography time-of-flight mass spectrometry (GC-TOF-MS) with its pivotal characteristics, including higher selectivity, resolution, sensitivity and accuracy is well-suited for the identification and quantitation of low molecular weight metabolites [29, 30]. In the present study, GC-TOF/MS-based untargeted metabonomics approach was utilized to profile metabolites in ESCC plasma samples from the pCR and non-pCR patients.

The major findings of this study were summarized as follows: i) A total of five key metabolites associated with ESCC chemoradiosensitivity were identified; ii) Among the five differential metabolites, isocitric acid, linoleic acid and citric acid were upregulated, while L-histidine, 3′4 dihydroxyhydrocinnamic acid were downregulated (Fig. 1b-f); iii) Multiple metabolic pathways, especially citrate cycle (TCA cycle), glyoxylate and dicarboxylate metabolism were significantly altered between pCR and non-pCR (Fig. 3 and Table 2).

In the previous study, altered metabolic profile was found between the pCR and non-pCR ESCC patients in the serum level detected by gas chromatography mass spectrometry (GC/MS) analysis and liquid chromatography mass spectrometry (LC/MS) analysis [21]. But only three differential metabolites (arabitol, uracil and 3-aminoglutaric acid) were detected by GC/MS analysis. While in the present research, five differential metabolites, including citric acid and isocitric acid were identified as plasma biomarker candidates for chemoradiosensitivity applied by GC-TOF/MS analysis. There is no evidence that plasma or serum was superior to be applied in clinical metabolomic study. However, there are indeed differential distributions of specific metabolites between plasma and serum. For instance, erythritol, glycerophosphocholines, glutamine, creatinine, and hexadecanoic acid in plasma, but not in serum, were shown to link with life expectancy for small-cell lung cancer patients [31]. In addition, another reason including chemical diversity and pretreatment of sample may also have impact on the disparate results. For example, citrate and citrate cycle metabolites were found in relative lower value in serum, partially because citrate in serum forms divalent cation complexes, which will precipitate by methanol extraction method [32]. Our study provided another dimension in investigation of the metabolites associated with nCRT respondence in the plasma level.

In the present study, L-histidine was found downregulated in the pCR patients. Previous study showed that increasing serum level of histidine was observed in breast cancer patient and strongly related with disease relapse [33]. Moreover, histidine degradation was found to be able to enhance the sensitivity to cancer therapy [34]. Meanwhile, recent study reported that high expression of FAM83A predicted a poor prognosis in lung adenocarcinoma patients, and histidine metabolism pathway was found significantly activated in FAM83A high expressed lung adenocarcinoma sample [35]. It is considered that the marked gain of histidine may enhance the total antioxidant and metal-binding capacity of the proteome of the cancer cell and thus potentially serve as a nonspecific compensatory mechanism to relieve consequences of the cancer-related aggravation of oxidative stress [36]. Furthermore, according to the pathway analysis, the tricarboxylic acid (TCA) cycle and the glyoxylate and dicarboxylate metabolism were found significantly related to ESCC chemoradiosensitivity. The TCA cycle is a key process for energy generation, which consumes oxygen and generates high amounts of ATP through oxidative phosphorylation. While for cancer cells, glycolysis is the primary pathway for energy production even if oxygen is available. Glycolytic pyruvate prefers to be converted into lactate, rather than enter into the mitochondrial TCA cycle. This phenomenon is called aerobic glycolysis or the Warburg effect. This kind of aberration has been proved to afford biosynthetic precursors for rapid macromolecule synthesis, and to keep cellular redox homeostasis for better survival [37, 38]. Mounting studies have demonstrated that there is a relationship between aerobic glycolysis and the occurrence of tumor drug resistance. And several key proteins in the glycolytic pathway have been discovered as promising targets for overcoming chemoresistance [39–41]. It was reported that suppressing glycolytic enzymes by inhibiting RAC1 showed reduced cisplatin resistance in esophageal squamous cell carcinoma [42]. Meanwhile, pretreatment whole-body total lesion glycolysis was uncovered as an independent predictor of outcomes in patients with esophageal cancer treated with definitive chemoradiotherapy [43]. In addition, altered glyoxylate and dicarboxylate metabolic pathway have been reported in various cancer. Evidence showed that glyoxylate and dicarboxylate metabolism was associated with the loss of tumor cell differentiation in lung adenocarcinomas [44]. Current study suggested that glyoxylate and dicarboxylate metabolism combined with other pathways could distinguish tumor from normal tissues in colorectal cancer [45]. In gastric cancer, glyoxylate and dicarboxylate metabolism were observed in the chromosomal instability type alone [46]. While previous study demonstrated that chromosomal instability is a favorable predictor of response to cisplatin-based neoadjuvant chemotherapy in patients [47]. In combination with the findings in this study, citric acid and isocitric acid, as the intermediates of the TCA cycle and the glyoxylate and dicarboxylate metabolic pathway, were up-regulated in the pCR patients. It is suggested that the energy metabolism was involved in the regulatory mechanism of sensitivity to neoadjuvant therapy for esophageal cancer.

There also exist some limitations in this study. The primary limitation is that this is a single–center study with relatively small number of subjects, making the data less conclusive. In addition, due to the restriction of the conditions, we only applied one metabolomics method to identify the potential changed metabolites. Furthermore, the predictive precision of those differential metabolites needed external validation. Larger samples and a combination of research methods may help us better understand the mechanism of ESCC chemoradiotherapy resistance.

## Conclusion

Overall, we found that significant alteration of metabolites between the responders and non-responders ESCC patients who received neoadjuvant chemoradiotherapy. The pCR group exhibited higher level of isocitric acid, linoleic acid, citric acid, and lower level of L-histidine, 3′4 dihydroxyhydrocinnamic acid than the non-pCR group. Changes in plasma metabolic signature may reflect reprogramming of the aforementioned metabolic pathways. Further study is needed to validate these findings using larger samples and to explore the underlying mechanism of ESCC chemoradiotherapy resistance.

## Supplementary information

**Additional file 1.**

**Additional file 2: Supplementary Figure 1.** Metabolic network of the changed metabolites and altered metabolic pathways in KEGG general metabolic pathway map. Red dots represent the increased metabolites in pCR group; Blue dots represent the specifically decreased metabolites in pCR group.

## Data Availability

All the necessary materials can be found in the text or supplementary materials. Due to the privacy policy, the confidential data materials could only be obtained with the permission of the corresponding authors.

## Abbreviations

AUC: Area under the curve
EC: Esophageal cancer
ESCC: Esophageal squamous cell carcinoma
GC-TOF/MS: Gas chromatography time-of-flight mass spectrometry
GC/MS: Gas chromatography mass spectrometry
LC/MS: Liquid chromatography mass spectrometry
nCRT: Neoadjuvant chemoradiotherapy
pCR: Pathological complete response
ROC: Receiver operating characteristic
TCA: Tricarboxylic acid
TNM: Tumor-node-metastasis
