# Supplementary figures and images for: Alteration of plasma metabolites associated with chemoradiosensitivity in esophageal squamous cell carcinoma via untargeted metabolomics approach
Source: BMC Cancer. 2020 Sep 2;20:835. doi: 10.1186/s12885-020-07336-9 (PMC7466788; doi:10.1186/s12885-020-07336-9)

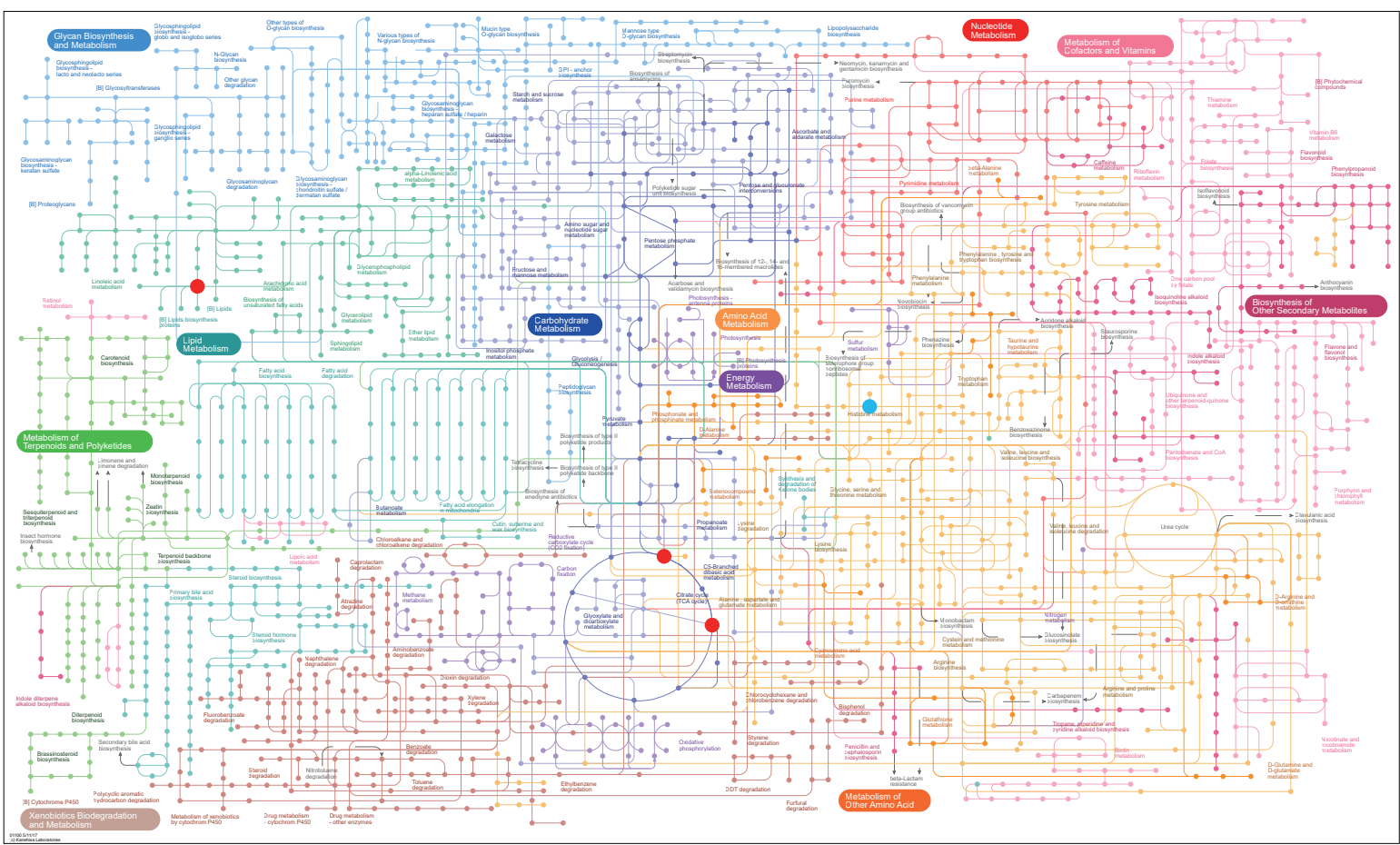

Supplement: Supplementary file 2 — Additional file 2: Supplementary Figure 1. Metabolic network of the changed metabolites and altered metabolic pathways in KEGG general metabolic pathway map. Red dots represent the increased metabolites in pCR group; Blue dots represent the specifically decreased metabolites in pCR group. [file 12885_2020_7336_MOESM2_ESM.pdf]
